# Lipid Transfer Proteins (LTPs)—Structure, Diversity and Roles beyond Antimicrobial Activity

**DOI:** 10.3390/antibiotics10111281

**Published:** 2021-10-21

**Authors:** Vinícius Costa Amador, Carlos André dos Santos-Silva, Lívia Maria Batista Vilela, Marx Oliveira-Lima, Mireli de Santana Rêgo, Ricardo Salas Roldan-Filho, Roberta Lane de Oliveira-Silva, Ayug Bezerra Lemos, Wilson Dias de Oliveira, José Ribamar Costa Ferreira-Neto, Sérgio Crovella, Ana Maria Benko-Iseppon

**Affiliations:** 1 Bioscience Centre, Genetics Department, Universidade Federal de Pernambuco, Recife 50670-420, Brazil; vinicamad@gmail.com (V.C.A.); liviambvilela@gmail.com (L.M.B.V.); olima.marx@gmail.com (M.O.-L.); mirelisantana@gmail.com (M.d.S.R.); r.s.roldanfilho@gmail.com (R.S.R.-F.); ayug.lemooos@gmail.com (A.B.L.); wilsondias.wo@gmail.com (W.D.d.O.); netocostaferreira@gmail.com (J.R.C.F.-N.); 2 Department of Advanced Diagnostics, Institute for Maternal and Child Health-IRCCS, Burlo Garofolo, 34100 Trieste, Italy; carlos.biomedicina@gmail.com; 3 General Microbiology Laboratory, Agricultural Science Campus, Universidade Federal do Vale do São Francisco, Petrolina 56300-990, Brazil; lane.roberta@gmail.com; 4 Department of Biological and Environmental Sciences, College of Arts and Science, Qatar University, Doha 1883, Qatar; crovella@qu.edu.qa

**Keywords:** cationic peptides, bioinformatics, prediction directed analysis, omics databases

## Abstract

Lipid transfer proteins (LTPs) are among the most promising plant-exclusive antimicrobial peptides (AMPs). They figure among the most challenging AMPs from the point of view of their structural diversity, functions and biotechnological applications. This review presents a current picture of the LTP research, addressing not only their structural, evolutionary and further predicted functional aspects. Traditionally, LTPs have been identified by their direct isolation by biochemical techniques, whereas omics data and bioinformatics deserve special attention for their potential to bring new insights. In this context, new possible functions have been identified revealing that LTPs are actually multipurpose, with many additional predicted roles. Despite some challenges due to the toxicity and allergenicity of LTPs, a systematic review and search in patent databases, indicate promising perspectives for the biotechnological use of LTPs in human health and also plant defense.

## 1. Introduction

Membrane biogenesis depends on the movement of lipids from their sites of synthesis (e.g., the endoplasmic reticulum) to other organelles, such as chloroplasts and mitochondria. In the course of the search for transport proteins, lipid transfer proteins (LTPs) were discovered in plant species, being recognized as important molecules. The first LTP in higher plants was described 46 years ago in potato (*Solanum tuberosum*) by Kader [1]. The name LTP was given due to the role of the newly discovered peptide in facilitating the transfer of phospholipids and galactolipids between a donor and an acceptor membrane during in vitro assays [2]. Also known as nsLTPs (non-specific lipid-transfer protein) or pLTPs (plant lipid transfer proteins), peptides of this group comprise a tunnel-like hydrophobic cavity capable of accommodating lipids [3] and other ligands, depending on their chemical nature [4]. It is believed that many of the functions performed by LTPs are associated with their ability to bind and transport lipids and other hydrophobic molecules.

This review addresses some little explored aspects of nsLTPs, including classification, structure, isolation and production methods, bioinformatics annotation and predicted roles. A literature search on potential technological applications (including patents) closes the review, also addressing perspectives and challenges on the biotechnological uses of these interesting plant peptides.

## 2. Classification, Nomenclature and Structural Features

The nomenclature of nsLTPs has been controversial due to a lack of consistent guidelines and standardization. This makes it very difficult, time-consuming and sometimes frustrating to compare data from different research groups [5]. Initially, nsLTPs were classified based on their molecular mass into nsLTP I and nsLTP II, with a relative molecular mass of 9 to 7 kDa, respectively. They are distinct in primary sequence identity (less than 30%) and lipid transfer efficiency [2]. Their primary sequences were characterized by an 8-cysteine conserved motif (8CM) (C-Xn-C-Xn-CC-Xn-CXC-Xn-C-Xn-C) which performs an important role in their 3D structure [6]. However, this method excluded other identified proteins that exhibited substantial homology to the nsLTPs. These proteins formed a new group called type III nsLTPs, which differs from types I and II by the number of amino acid residues present in the 8 CM framework intervals [7]. Another classification system was proposed by Boutrot et al. [8], based on phylogenetic studies. This approach categorized nsLTPs into nine groups (type I-IX).

Later studies applied this classification system to different species with slight modifications [9,10,11], adding group X [9] and a later grouping XI [12,13] not reported before. This classification system is currently the most used in approaches that combine sequence alignment, phylogeny and structural biochemistry. However, this system is applicable to angiosperms, but largely excludes nsLTPs species of non-flowering plants, due to limited sequence homology. This led to a new classification of five major nsLTP types (nsLTP1, nsLTP2, nsLTPc, nsLTPd, and nsLTPg) and five minor types with fewer members (nsLTPe, nsLTPf, nsLTPh, nsLTPj, and nsLTPk) [14]. Additionally, this classification is not based on molecular weight, but on the position of a conserved intron, amino acid sequence identity and spacing between cysteine (Cys) residues.

Salminen et al. [5] proposed another format, naming the nsLTPs as follows: AtLTP1.3, OsLTP2.4, HvLTPc6, PpLTPd5, and TaLTPg7 where the first two letters indicate the plant species (e.g., At = *Arabidopsis thaliana*, Pp = *Physcomitrella patens*, etc.), LTP1, LTP2, LTPc indicate the type. Finally, the last digit (here 3–7) indicates the gene/protein number within a particular type of LTP. For clarity, in the case of LTP1 and LTP2, it is recommended to include a punctuation mark between the type specification and the gene/protein number, which is not necessary for LTPc, LTPd, LTPg and other LTP types defined with a letter.

The expanded classification system contributed significantly to the general development of classification methods. However, further improvements can be made to increase the understanding and robustness of the system, including nsLTPs of lower plants and also considering important signatures that define their function or specificity. In view of the growing volume of available omics databases, it is important to periodically revisit nsLTP classification proposals, combining genomic, transcriptional, and three-dimensional structure analysis, thus designing a classification system that combines evolutionary and functional data.

### 2.1. Conserved Domains and Motifs

**Figure 1:**
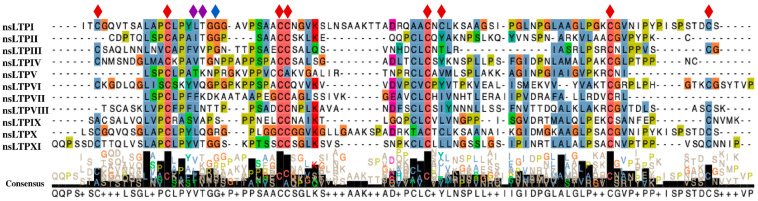
Alignment of representative sequences of each nsLTP type. Diamonds (above) indicate regions of conserved amino acid residues, red for 8M motif cysteines, blue for glycine and purple for residues contributing to helix spacing. The Clustal X colour scheme highlights conserved amino acid residues besides their physicochemical characteristics. The black bar below refers to the conservation between all aligned sequences with their respective amino acids. The consensus sequence is presented at the bottom of the figure, where the “+” signs indicate high variable positions.

The most conserved region is the 8CM motif. The main difference between the nsLTP types is defined by the nature of the disulfide bridges, with greater variation in cysteines 5 and 6 (C5 and C6), leading to functional effects in the tertiary structure. However, this is not the only important region, as some amino acids seem to distinguish type I nsLTPs from the others, such as the conservation of a glycine between helix 1 and helix 2, connected by the disulfide bridge between C2 and C3. Greater distances between helix 1 and helix 2 were verified in all nsLTP sequences where this glycine between the helices was mutated. Besides, other lysine and tyrosine residues are especially conserved among type I nsLTPs (Figure 1, diamond on top of alignment) [13]. These differences contribute to particular structures and can directly interfere with their mechanisms of action.

### 2.2. Tertiary Structure and Available Models

**Figure 2:**
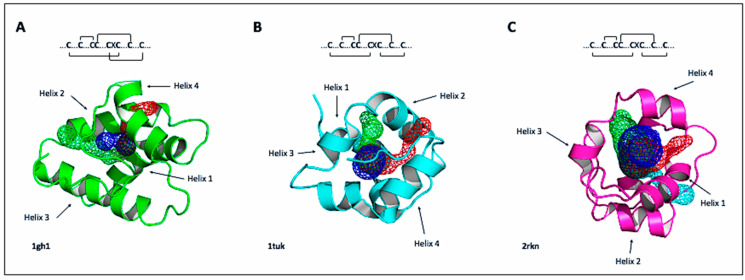
Three-dimensional representation of nsLTPs: (**A**) 1GH1 (nsLTP type I, in green), (**B**) 1TUK (nsLTP type II, in cyan) and (**C**) 2RKN (nsLTP type IV, in magenta). At the top is the disulfide bridge pattern of the respective structures, where C represents cysteine and X any amino acid. The black arrows indicate the four helices present in the nsLTP structures. The mesh spheres (blue, green and red) in the center of the structures represent the hydrophobic pocket identified by Caver 3.0 PyMol plugin (https://github.com/loschmidt/caver-pymol-plugin/blob/master/COPYING), public license [20] and visualized in PyMOL.

The three-dimensional structure of nsLTPs is characterized by the presence of a compact domain generally composed of four helices (H1–H4) connected by short loops (L1–L3), besides an unstructured C-terminal tail (Figure 2).

The second and third helices and the C-terminal loop are usually longer in type I than in other types of nsLTPs. The domain is held tightly together by a large number of intramolecular hydrogen bonds, as well as four cysteine disulfide bridges conserved with two patterns: (1): C1-C6, C2-C3, C4-C7 and C5-C8 for Type I nsLTPs [15] and (2): C1-C5, C2-C3, C4-C7 and C6-C8 for type II [16] and IV [17].

These three-dimensional structures present a large tunnel-like internal cavity that accommodates different types of lipids and also exhibit unusual stability against thermal and digestive processing [9]. These folds provide a different lipid binding specificity at the nsLTP binding site, being the structure of nsLTP II relatively more flexible and with less lipid specificity when compared to nsLTP I [18].

The first three-dimensional structure of an LTP was based on 2D and 3D 1H-NMR data in an aqueous solution of LTP1.1 (Protein Data Bank, code PDB identifier ID: 1GH1) purified from wheat seeds (*Triticum aestivum*) [15,19]. Currently, several three-dimensional structures of nsLTPs have been determined, either by NMR or X-ray crystallography, either in its free, unbound form or in a complex with ligands.

## 3. Roles of nsLTPs

### 3.1. Defense

One of the most reported functions for nsLTPs regards plant defense against pathogens. Besides experimental data indicating an antimicrobial action, their size, molecular structure, and the presence of cysteines in conserved positions are characteristic of AMPs and pathogenesis-related protein of the PR-14 superfamily [21,22], where they have been classified. Finkina et al. [23] suggested that the defense response mechanism probably involves the secretion of nsLTPs into the apoplast, allowing them to bind to other lipid molecules secreted by plants (such as jasmonic acid) or to molecules secreted by pathogenic microorganisms.

**Figure 3:**
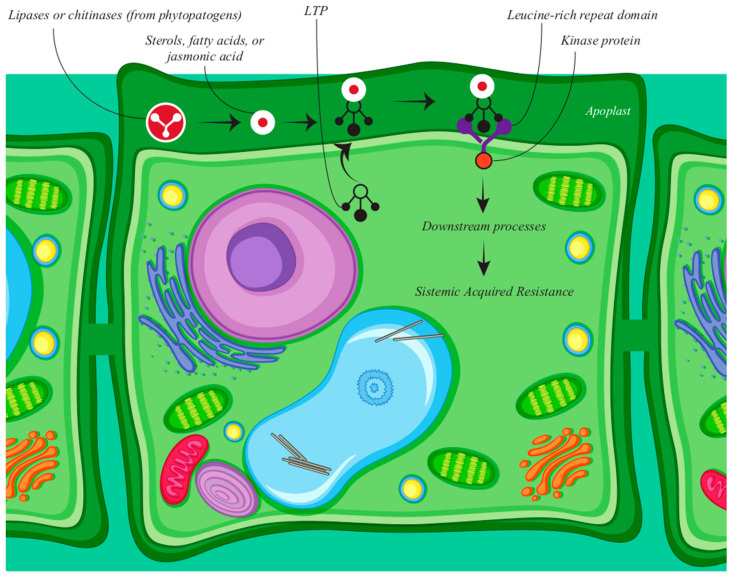
Schematic representation of the defense response and systemic acquired resistance events including the participation of LTPs in a plant cell.

In this sense, nsLTPs bind to lipid molecules and interact with receptors such as serine/threonine protein kinases that contain an extracellular leucine-rich repeat (LRR) domain, besides a transmembrane region and a cytoplasmic protein kinase (PK). This interaction activates a versatile second messenger-mediated signal transduction and causes a mitogen-activated cascade of protein kinases (MAPK), inducing transcription factors, protective factors, PR (pathogenesis-related) proteins, including other AMPs, finally inducing SAR (acquired systemic resistance) as illustrated in Figure 3.

The role of nsLTPs in relation to biotic stresses is well established in the literature, either by direct mechanisms, such as acting as antimicrobial agents, as lipid transporters, or indirectly as signaling molecules [4], as discussed below. Therefore, plant nsLTPs have become the target of studies involving genetic transformation, aiming at acquiring resistance to pathogens, besides their potential use for drug development, given their potential activity against human pathogens and inhibition of clinical sepsis [24], as we will discuss later.

### 3.2. Growth, Development and Environmental Adaptation

In addition to their role in defense, nsLTP are associated with biological processes such as seed growth and germination [23,25], fruit softening and ripening [26], among others. In this sense, it is understood that plant cells are able to expand their internal volume through turgor, whereas the extension of the cell wall is the factor that limits this process [27]; therefore, nsLTPs can be described as proteins that ‘relax’ or ‘loosen’ the cell wall [28]. Such a role justifies the association of nsLTPs also to abiotic plant stress. For example, a study conducted by Hairat et al. [29] showed that the overexpression of two wheat nsLTPs (genes *TaLTP40* and *TaLTP75*) in *A. thaliana* increased the tolerance of transformed individuals to salt stress. A similar study conducted by Yu et al. [30] generated transgenic *A. thaliana* plants by overexpression of *TaLTPIb.1*, *TaLTPIb.5*, and *TaLTPId.1* genes with increased cold tolerance.

Research by Hairat et al. [29] showed that the transcriptome of vegetative and reproductive tissues (except ovaries and roots) of *T. aestivum* expressed *TaLTPs* in response to stresses such as cold, salt and drought. Another study by Fang et al. [31] identified 22 modulated nsLTPs genes from *T. aestivum* under different abiotic stresses such as salinity and drought. Similarly, Xu et al. [32], showed in their study with tobacco (*Nicotiana tabacum*) that nsLTPs increased tolerance to stresses such as drought and salt.

### 3.3. Other Roles

It is known that nsLTPs are involved in the assembly process of lipid barriers [3,23]. However, the transport mechanism of lipid components for cuticle formation is not properly known, with open questions on the precise role that nsLTPs play in cuticle structuring [33]. In this sense, it is hypothesized that the nsLTPs of the G-type (nsLTPGs) act in the loading of wax for functional activities, such as sealing the vulnerable edge around cell junctions (cell-cell interface) as well as in fortifying the cell wall and promoting cuticular wax deposition [33]. As a result, a role of nsLTPGs with biosynthesis and accumulation of suberins, cuticular waxes and sporopolelins has been proposed [34]. This association has been confirmed by their location in plants since nsLTPs are found in the cutinal lining of organs such as leaves, stems and flowers. In this sense, type II nsLTPs are more often associated with the coating of organs (especially underground) with suberin [23], besides association with the formation of sporopolelin barriers and cuticular waxes [3,35].

Another proposed function regards the proapoptotic activity, which is based on the similarity between nsLTPs and the human BID protein. Both have similar structural signatures, which is considered indicative of interactions with membrane lipids. The pro-apoptotic protein BID acts in the cytosol in the presence of lysophospholipids generated during apoptosis, affecting the mitochondria and causing the release of apoptotic factors (including cytochrome c). Similarly in maize (*Zea mays*), in the presence of lysophospholipids, nsLTPs also induce the release of cytochrome c from the mitochondria. Thus, lysophospholipids modify membrane properties, facilitating the action of other pro-apoptotic proteins [36]. This hypothesis of proapoptotic function is reinforced by the fact that both peptides (BID and nsLTPs) comprise an internal cavity capable of binding and transferring similar lipids [37].

**Figure 4:**
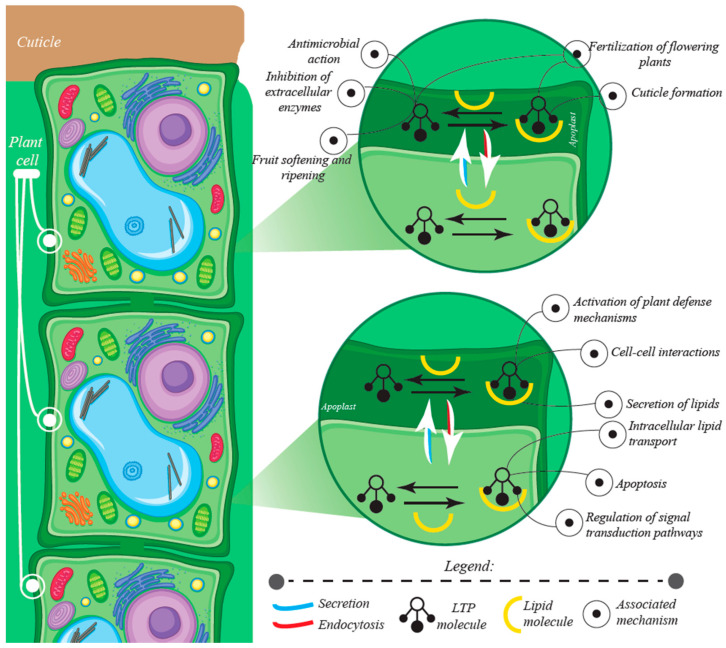
Illustration of possible activities performed locally by lipid transfer proteins in the cell interior, including apoptosis, intracellular lipid transport, regulation of signal transduction pathways, and in the apoplastic route; fertilization, cuticle formation, antimicrobial activity, inhibition of extracellular enzymes, fruit softening and ripening, activation of plant defense mechanisms, cell-cell interactions, and secretion of lipids.

The roles and mechanisms of nsLTPs are illustrated in Figure 4.

It has also been suggested that nsLTP can transfer lipid molecules that play important roles in inducing defense signaling cascades by forming a sterol-elicitin complex that is perceived by the plasma membrane receptors. In this sense, recombinant tobacco type I nsLTP was able to form a complex with jasmonate, and to be recognized by elicitin receptors, inducing systemic resistance against the fungal pathogen *Peronospora parisitica* [38,39]. Another study showed that the gene *TaMs1* expressed nsLTP in *T. aestivum* presented higher induction when associated with acetic acid-2-indole (IAA) and abscisic acid (ABA) [40]. A study by Wu and Burns [41] showed that nsLTP genes are also up-regulated in ripe fruits during their evolution towards the fruit abscission.

## 4. Genomic Distribution and Macrosynteny

Plants have suffered many events of genome duplication during evolution. These events may likely have led to the emergence of new gene/protein features [42] giving new attributes to paralogs through neofunctionalization, dividing the original function (subfunctionalization) or even impairing gene expression by silencing [43] at a much amplified level compared to other eukaryotic groups. The genomic distribution of some gene families provides an insight in such groups, being a source of information that may have biotechnological potential, since plant improvement programs are mostly based on genomic mapping, also comparing data among different accessions or taxa, besides inferring about gene evolution itself [43,44].

nsLTPs are multifunctional ubiquitous proteins present in angiosperm families like Brassicaceae [44,45], Solanaceae [9], Poaceae [46], and Malvaceae [47], but also in mosses and ferns [14]. Thus, it is plausible to conjecture that duplication events played a crucial role in the evolution of these genes, once it is known that ancient polyploidization events occurred before the diversification of seed plants and also before the emergence of the angiosperms [48]. Thus, genome-wide analysis can shed light on these questions.

A recent genome-wide study analyzing all classes of PR proteins in cassava (*Manihot esculenta*) in response to whitefly (*Aleurotrachelus socialis*) identified 447 *PR* genes from which 30 were described as *nsLTPs* distributed among 12 (out of 18) chromosomes. This study showed a clusterization and tandem organization for most of these genes, including *LTPs* [49], suggesting that tandem duplications have played an important role in the expansion and diversification of the whole class, once the same phenomenon can be observed for other PR proteins in different plants [50,51,52].

In tomato (*Solanum lycopersicum*, Solanaceae), the majority of the nsLTPs mapped in the chromosomes were organized in clusters. Of all nsLTPs, 60%, out of 64 *nsLTP* were distributed along 7 out of 12 chromosomes. The chromosomes 3 and 8 concentrated the highest number of genes (15 each) and the biggest clusters (six genes each). Some tandem duplications and minor clusters were pointed in chromosomes 1, 3, 6, 8, and 10, whereas chromosome 12 had only one gene mapped. Together with the expression data this information provides a source to understand the regulatory aspects of nsLTPs since, for example, in chromosome 3 a cluster included genes expressed exclusively in roots [53].

In a comparative study, Ji et al. [54] found 89 syntenic *nsLTPs* orthologues between *A. thaliana* and *Brassica oleracea*. Analyzing the genome of *B. rapa* and also comparing with *A. thaliana*, Li et al. [12] found 63 genes in colinear conserved blocks following the duplication events suggested for these taxonomic groups. Furthermore, this pattern was evident also in Poaceae, where a study in barley identified 70 *nsLTPs*, from which 36 were arranged in 15 distinct tandem duplicated repeat blocks in seven chromosomes [55]. By comparing members of the two taxonomic families (Poaceae and Brassicaceae) Boutrot, Chantret, and Gautier [8] identified 26 tandem duplication repeats out of 52 loci in rice (*Oryza sativa*) and 18 out of 49 loci in *A. thaliana*. The same study found 122 putative *nsLTPs* in wheat (*T. aestivum*) and suggested that the expressive amount of genes in this crop is caused by duplication events and the remarkable polyploidy, corroborating the evolutive history of the group, and confirming events that led the multi-functionalization of the *nsLTPs*.

Briefly, this great diversity and apparent redundancy of nsLTPs in angiosperm genomes is in line with the neofunctionalization process that led to the emergence of paralogs and multiplicity of functions (discussed below). Furthermore, judging by the available evidences, the distribution of genes is not random, given the significant conservation among different species and the existence of gene clusters associated with tissue-specific expression patterns.

## 5. Transcriptomics and Gene Modulation

Studies related to *nsLTP* expression generally focus on biotic stress responses, showing important achievements. However, as mentioned before, this is not the only function of nsLTPs. Also, the mentioned duplication events led to a high diversification allowing the emergence of other predicted functions for *nsLTPs* that may be confirmed by analyzing their expression profile [33]. Thus, an understanding of the expression aspects of this gene group is important to go deeper into their biochemical and physiological attributes, also involving biotechnological manipulation as molecular breeding and transgenics [56,57,58]. The expression pattern of *nsLTPs* is quite diverse and there are reports of their detection in most plant tissues and stages, as verified in studies carrying different transcriptomic approaches, from microarray to RNA-Seq as well as real-time quantitative PCR (RT-qPCR) analysis. These studies indicate a multi-functional action of these proteins in molecular signaling, plant development, biotic and abiotic stresses [56,59,60,61].

The role of the nsLTPs in response to biotic stress stimulus is well reported, triggered by the main disease-causing agents in plants like bacteria [62], fungi [25], viruses [63], nematodes [64] and insects [65]. Therefore, they are frequently referred to as members of the pathogenesis-related proteins—14 (PR-14) superfamily [66].

A study performed using the northern blot approach evidenced the distinctive functional characteristics of *Capsicum annuum* nsLTP I and II (CaLTP I and II) in transgenic tobacco (*N. tabacum* cv. Xanthi). It has shown that these nsLTPs enhanced the resistance of tobacco to pathogens *Phytophthora nicotianae* and to *Pseudomonas syringae*. Also, the mentioned genes regulated the expression of other PR-proteins, as PR-4 was constitutively expressed in association to the overexpression of *CaLTP II*, indicating a role of nsLTP in long-distance systemic signaling [67].

Expressed Sequence Tags (ESTs) and SuperSAGE (Super Serial Analysis of Gene Expression) screenings in *Glycine max*, *Medicago truncatula*, *Arabidopsis thaliana* and *Brassica napus* retrieved hundreds of PR-14 transcripts and linked their expression to biotic and abiotic stresses, in soybean. For example, SuperSAGE tags analysis indicated that PR-14 was induced under drought and the Asian rust-causing fungus *Phakopsora pachyrhizi*, while in canola unigenes were detected in seed and leaf [52,68].

The evolutionary and regulatory relations between nsLTP in rice and wheat were traced using microarray and RT-PCR analysis. This approach revealed at least 15 conserved cis-regulatory motifs that guide the expression of *nsLTP* genes in these species. Moreover, the study evidenced that mutations on these elements also directed the expression and functional diversity of *nsLTPs* [46,69]. Together with chromosome mapping data, especially by comparing chromosomes 11 and 12 of rice with those of other Poaceae, indicated that the multi-functionality of nsLTP is due to a plethora of events that range from mutations in the regulatory regions to segmental duplication events and polyploidization during speciation in Poaceae [10,46,69,70].

Another study approached the transcriptional modulation of AMPs (including nsLTPs) in maize (*Zea mays*) using RT-qPCR. The results showed the induction of nsLTPs when the plant was inoculated with *Spodoptera frugiperda* and *Aspergillus flavus* [71]. By a similar approach, Wang et al. [64] elucidated the role of the CsLTP2 from cucumber (*Cucumis sativus*) involved in the response against the root-knot nematode (*Meloidogyne incognita*) under the regulation of the MYB transcription factor (TF). The authors suggested that TF-MYB may be an important regulator of the transcriptional hub of *nsLTP II* genes involved in the response against this nematode. Kido et al. [57] described a novel nsLTP from sugarcane (*ScLTP*) induced in the presence of methyl jasmonate and repressed in response to salicylic acid. This *ScLTP* gene was also involved in the adaptative reaction to low temperatures and drought stress [59].

A survey via RNA-Seq and RT-qPCR in tomato (*S. lycopersicum*) indicated 13 tissue-specific *nsLTPs* transcripts in bud and root tissues. Additionally, a correlation was observed between organized chromosome clusters and the accumulation of suberin in roots [5]. Some gene clusters presented high expression in buds and flowers, indicating a role in another development, pollen formation, generation of pollen exine, pollen tube growth and germination [5,33,53].

Considering all evidence, the multifunctionality of nsLTPs (Table 1) is probably a consequence of their participation in the transport of different lipid proteins important to plant survival and adaptation to different environmental conditions in the course of evolution.

## 6. Identification and Isolation

The isolation of biomolecules is achieved by applying purification methods compatible with their biochemical properties. Table 2 lists isolated native nsLTPs and their tested biological activities. It is possible to observe that most nsLTPs were isolated from seeds, a structure that shelters the embryo surrounded by a rich reserve of nutrients, including lipids.

Purification of nsLTPs is initiated by their extraction from cellular tissues or extracellular fluids (e.g., nectar and latex). Plant organs that have a high fat or oil content, such as seeds, can be pretreated to remove lipids that might interfere with the purification of nsLTPs. An alternative is to decrease the crushed material by leaving it immersed in a non-polar solvent (e.g., hexane or petroleum ether) prior to extraction [84,92]. Based on the fact that nsLTPs are basic (have a positive net charge) [2,23,53] their extraction is performed in saline buffer or acid solution, prioritizing their stability or dissolution, respectively. After this process, nsLTPs can be precipitated by regulating the pH of the crude extract based on the isoelectric point of the peptide or by fractionation in ammonium sulfate, the latter being at a high relative saturation value (approximately 80% of the salt) [77,82,89,92].

Most successful approaches for isolating nsLTPs (Table 1) involve chromatography. They usually start with an ion-exchange separation (e.g., DEAE-Sephadex or Q Sepharose column) in a way that the charged matrix will adsorb (or not) the net positively charged peptide. Another alternative for starting purification is size exclusion chromatography on a gel filtration column (e.g., Sephadex G-50 column), where the matrix separates the small nsLTP from the larger proteins present in the same sample. Finally, purification is completed by reverse-phase high-performance liquid chromatography (RP-HPLC), in which the nonpolar amino acid residues of nsLTPs adhere to the hydrophobic chain matrix (ranging from C_4_ to C_18_). These combinations ensure yields of 10 to 773 mg of nsLTPs per kilogram of seeds [77,87,90,91] and 4.2 mg of nsLTPs per gram of extracted proteins [85].

In addition to chromatography, nsLTPs can be isolated by one-dimensional polyacrylamide gel electrophoresis (1-D SDS-PAGE) [81,82]. However, the biological activity of the obtained peptide is compromised by the denaturation of its structure by the detergent SDS, leaving only its sequence and structure prediction for analysis. For this analysis, first, bands with different molecular weights are excised from the gel and the retained proteins are cleaved with proteases to identify their sequences by high-performance liquid chromatography tandem mass spectrometry (LC-MS/MS). Next, characteristics such as conserved domain, secondary structure and prediction of tertiary structure are evaluated in silico to prove whether the isolated candidate sequences belong to the nsLTPs [81,82].

The molecular mass of isolated nsLTPs varies between 9 and 10 kDa, determined by SDS-gel electrophoresis, gel filtration and mass spectrometry. However, non-canonical nsLTPs may have a smaller size variation, such as Ace-AMP1, isolated from onion (*Allium cepa*) with molecular mass 7.5 kDa [77]. Regarding the pI of the isolated peptides, values between 7.8–8.5 [82,83,85] have been observed. Isoforms can be detected by two-dimensional electrophoresis, distinguishing each one by its pI [83]. In parallel, the binding activity of nsLTPs to lipids is identified by the in vitro fluorescence assay. The formation of a lipid-nsLTP complex promotes the crystallization of the peptide structure and amplification of the intrinsic fluorescence of its aromatic residues (e.g., tryptophan) [93,94]. For instance, Wang et al. [89] and Lin et al. [80], observed an increase in the intrinsic fluorescence intensity of nsLTPs isolated from *Phaseolus mungo* and *Brassica campestris* when exposed to the lipid lyso-α-lauroyl-phosphatidylcholine (Lyso-C_12_).

This method is also suitable for evaluating the functionality of recombinant nsLTPs, as performed by D’Agostino et al. [53] who observed an increase in intrinsic fluorescence of nsLTPs from tomato (*S. lycopersicum*) (Sola I 3) when binding to 1-palmitoyl- 2-lysophosphatidylcholine (Lyso-C16), showing a dissociation constant of 85.5 ± 6.

Another alternative for monitoring lipid-binding activity is through fluorescent probes such as 6-(p-Toluidino)-2-naphthalenesulfonic acid (TNS). The fluorescence intensity of TNS is increased by its binding to the hydrophobic cavity of nsLTPs. Therefore, the interaction between nsLTPs and lipids can be observed by reducing the fluorescence intensity of the nsLTP-TNS complex, as the lipids compete for the binding site.

In pea (*Pisum sativum*) seed recombinant PsLTP I, it was observed that unsaturated fatty acids (linoleic and linolenic acids) and negatively charged lysolipids (lyso-myristoyl phosphatidylglycerol and lyso-palmitoyl phosphatidylglycerol) had higher efficiency of binding to the hydrosephatidylglycerol cavity, since the intensity of the PsLTP I-TNS complex was reduced to 23% [90]. In turn, dill (*Anethum graveolens*) antifungal AgLTP presented a greater affinity for jasmonic acid and lyso-palmitoyl phosphatidylglycerol [78]. It can be noted that the affinity is related to the geometry of the lipid as well as the polarity of the phosphate group. Such factors are crucial for lipids to fit in the peptide cavity [78].

The isolation of nsLTPs is usually performed from their native form but often the isolation of a single purified nsLTP is a very laborious task and low amounts are obtained. On the other hand, genes encoding these peptides can be identified in the genome (DNA) or as an expressed product (messenger RNAs, mRNAs) [53,78,81,90]. In the case of isolated total mRNA, conversion to cDNA is performed by reverse transcription and the sequences are amplified by PCR [53,90]. The DNA and cDNA sequences encoding nsLTPs, evaluated by computational tools, are then cloned into appropriate vectors to be inserted into host systems [53,78,81,90].

## 7. In Silico Identification of nsLTPs in Data Banks

For the search in omics databases, an accurate understanding of the sequences of interest is necessary. This ‘mining process’ generally demands accurate information about the desired molecular targets. In this sense, information such as conservation patterns and/or sequence probes (so-called ‘seed-sequences’) are commonly used for retrieving information from databases, since direct keyword searches may yield uncured undesirable results. As with most AMPs [95], identification requires additional steps due to the great variation that these sequences present. The most traditional search regards the Basic Local Align Search Tool (BLAST), a method that allows measuring local similarity through a score by maximal segment pair (MSP) to measure statistical significance [96]. This method generally retrieves only sequences with significant similarity to the seed-sequences used. A more suitable method involves the prediction of homologs using the probabilistic model, called the Hidden Markov Model (HMM). This search is based on the detection of sequence patterns with a sensitive algorithm, relying on the strength of their underlying probability models [97]. However, this method requires a prior analysis and definition of patterns since the available HMM-models for a given gene class do not always cover all the variability of sequences [95]. The last method differs from the previous ones in that it is based on the search for patterns with a Regular Expression (RegEx) algorithm which considers the conservation of motifs inherent to sequences using a search based on text structure, allowing the detection of a set of defined patterns [95].

As highlighted by Santos-Silva et al. [95], it is advisable to use a combination of the three approaches, as each can have different outputs. A curation of the obtained sequences is also advisable, in order to eliminate sequences from other gene classes with domains or signatures in common. In this sense, functional domain identification is an essential confirmation step, also helping to find the new molecules of interest, going through several tools using selector filters.

The most widely used domain identification methods consist of identifying conserved regions through predictors, as the tool CD-Search [98], which allows massive submission of batch targets (BATCH). By using local tools one can adjust search parameters to retrieve conserved regions in sequences previously identified in data banks [99].

This step starts the characterization of the primary structure, being followed by the secondary, which consists of confirming the equity of characteristics of the molecular targets, such as charge, pattern of disulfide bridges, structural motifs of the candidates, and properties such as isoelectric point, molecular weight, point of cleavage and prediction of antimicrobial activity [95].

**Figure 5:**
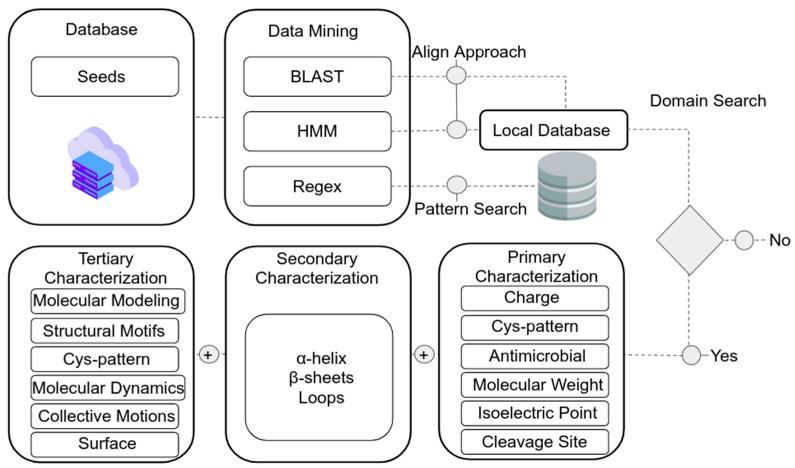
Flowchart with the steps required to identify nsLTP sequences from omics databases (nucleotide and amino acid sequences).

The characterization takes as a starting point the conserved domains for the prediction of the cleavage point using algorithms such as Deep Neural Network by the webserver SignalP5.0 [100]. In sequences the prediction and classification of ternary cysteine (free cysteines, a half-cystine or linked to the ligand) is evaluated using an algorithm based on Support Vector Machine (SVM) [101]. The calculation of the Isoelectric Point (pI) and the molecular weight (Da) can be performed using an Isoelectric Point Calculator (IPC) among other physicochemical characterizations also made possible by a web-server [102]. The general method for identifying nsLTPs in databases follows the workflow presented in Figure 5.

In addition, the antimicrobial properties of AMPs (including nsLTPs) can be explored by various algorithms by comparing sequences with databases such as CampR3 (Collection of Anti-Microbial Peptides) [103], a database with more than 4000 curated peptides, webserver prediction is based on algorithms such as Support-Vector Machine (SVM), Random Forest Classifier (RFC), Artificial Neural Network (ANN), and Discriminant Analysis Classifier (DAC). Another useful resource is ADAM (A Database of Anti-Microbial Peptides) [104] containing 7007 unique sequences and 759 experimentally validated structures, besides tools using SVM and HMM algorithms to infer sequence and structure of models of interest. In turn, the MLAMP data bank deserves mention, including 878 AMPs and 2405 non-AMPs (as negative controls), extracts and prediction for activities such as (1) antibacterial; (2) anti-cancer/tumoral; (3) antifungal; (4) anti-HIV and (5) antiviral. The predictions are based on an RFC algorithm [105]. A combined analysis of nsLTPs with tools of two or more of the mentioned data banks has an estimated collection of 126 LTPs sequences and can provide interesting evidence about predicted structure and function, especially regarding antimicrobial activity.

Another important task regards the conception of a three-dimensional structure of the protein. This step allows studying functional aspects inherent to the protein architecture like the chemical nature of allergenicity [106]. In this sense, comparative modeling based on previously described accurate models is the most recommended strategy, which can be done automatically or manually by the SWISS-MODEL webserver by the hybrid method (ProMod3) [107,108], or manually by the MODELLER software using the iterative method of optimization to satisfy spatial constraints [109]. This is not a simple task, since it requires careful criteria for model choice and validation in order to accurately assess stereochemistry and molecular folding.

Once a model is obtained, its confirmation takes place through molecular dynamics, in order to confirm its stability. Molecular dynamics is a controlled simulation method that allows evaluating the change of atomic coordinates in a system [95], allowing an approximation of a model to its native state. In this sense, the calculation of molecular dynamics is guided by an approximation of an empirical parameter, called force field [110]. Through this method, it is possible to confirm the three-dimensional and secondary structures of the model, as well as the integrity of hydrogen bonds and/or disulfide bridges associated with protein compaction. Furthermore, it is possible to ascertain the dynamic nature of the protein and identify rigid and flexible regions, besides the physicochemical nature of the model’s topology.

Such methodological approaches open promising prospects for so-called top-down analyses, i.e., nsLTPs mining in omics databases with further deep-characterization followed by laboratory evaluations/validations, allowing a broader view of their diversity and functional prediction. Steps for lab validation are presented in Santos-Silva et al. [95].

## 8. Heterologous Expression and Biotechnology

Biotechnological manipulation helped to circumvent some limits imposed by the isolation of native nsLTPs from plant tissues, stimulating several insights [111] and allowing functional tests. Based on a bibliographic search with the terms “nsLTP” AND “recombinant expression” and “nsLTP” AND “heterologous expression” until July 2021 in the National Center of Biotechnology Information’s PubMed database, 29 works were retrieved, including the heterologous production of nsLTPs. The approaches identified include diverse biotechnological techniques such as protein production by chemical synthesis, recombinant DNA production or heterologous expression, although the extraction from natural sources is still prevalent, as discussed above [32,81,112]. The other two ways of obtaining nsLTPs include some specific procedures according to the purpose of using nsLTPs.

One target regards the production of allergens, whereas the use of chemical synthesis is the best option, due to the easy incorporation of unnatural amino acids and more solid post-translational modifications compared to other strategies [113]. The synthesis of full length nsLTPs is complex and difficult due to patterns of conserved regions and disulfide bonds. So far, we are not aware of any bibliography reporting this synthesis. Due to this issue, a more viable option may be the synthesis of specific parts (smaller portions) of these AMPs that may have antimicrobial activity. However, the heterologous expression stands out due to the satisfactory cost-benefit ratio when the yield is taken into account. These allergenic *nsLTPs* may be expressed in different types of hosts such as bacteria, yeasts and plants, being important for clinical diagnostic tests.

Some limitations of heterologous expression have been reported, such as low yield, toxicity of the expressed protein to the host system, and difficulties in protein folding after post-translational modifications. However, most of these difficulties have been overcome with expression strategies as technological advances increase. Some measures can be taken, such as proper use of the host system, fusion proteins coupled to the coding region of the gene, tandem copies of the genes to be expressed, codon optimization, as well as promoter choice, in order to increase levels of transcription and protein expression [114,115]

An innovative expression strategy was used by Safi et al. [116], who analyzed the regulation of a wheat nsLTP gene. The promoter region of the TdLTP4 gene (*PrTdLTP4*) was fused to the β-glucuronidase (*gusA*) reporter gene and expressed in *A. thaliana* transgenic plants. As a result, the expression of *gus* was observed in arabidopsis leaves from 8 to 12 days of age, having considerably increased the transcript expression when subjected to stress with NaCl and mannitol. The expression of genes was analyzed and validated by histochemical tests and RT-qPCR, respectively. The data showed that *PrTdLTP4* worked as a promoter induced by abiotic stress in the heterologous system, which could help in future improvements for wheat harvest.

### 8.1. Expression in Plants

nsLTPs play fundamental roles in several biological processes in plants, ranging from the formation of cutin, to fighting pathogens, and triggering a cascade of signals in the plant’s defense. *nsLTP* I have transcribed genes corresponding to proteins located in peripheral aerial tissues that allow cutin formation [117]. Furthermore, the genes transcribed from *nsLTP* II are active in suberin-forming tissues such as roots [118]. Studies report that the regulation of *nsLTPs* gene expression in plants is complex and controlled by factors such as space and time [116], and may be related to biotic and abiotic stresses [25,116]. An example of abiotic stress regards a tobacco (NtLTP4) present in the extracellular matrix that increased tolerance to salt and to drought in transgenic tobacco, in addition to a relief from the toxic effects of Na+ and reactive oxygen species (ROS). This transgene also regulated the transpiration rate of the plant and interacted in response to stress mediated by mitogen activated protein kinases (MAPK) in tobacco [32].

Another study showed the activity of recombinant *nsLTPs* in *A. thaliana* and *Saccharomyces cerevisiae* under biotic stress [119]. In this study, a population of *A. thaliana* resistant to trichothecin (a type of mycotoxin that poses a risk for food safety in humans and animals) was analyzed based on the expression of *nsLTP* genes (*AtLTP4.4* and *AtLTP4.5*). The expression of these genes was induced 10 times more than the wild type. The overexpression of *AtLTP4.4* turned the *A. thaliana* plants more resistant to trichothecin than *AtLTP4.5*, a situation also observed in yeast in relation to wild strains. Both arabidopsis and yeast were subjected to treatment with trichothecin and there was an increase in ROS in wild types in both taxa, whereas the production of ROS was attenuated in the individuals that had overexpression of the *AtLTP4.4* gene. The results showed that trichothecenes cause ROS accumulation, whereas the overexpression of *AtLTP4.4* protected against oxidative stress, increasing the plant antioxidant response.

### 8.2. Expression in Bacteria

Due to the antimicrobial function of nsLTPs, heterologous expression is a challenge when using prokaryotic organisms as host vectors. Due to their inhibitory action, nsLTPs can exert a lethal effect on their production in the host microorganism, in addition to their susceptibility to undergo degradation by secreted proteases. However, several strategies may be used to circumvent this challenge, such as carrier or fusion proteins [90,114,120]. For the purpose of producing proteins by heterologous expression, *Escherichia coli* is the most used expression model, due to its ease of production and better cost-effectiveness [114,120]. Many nsLTPs have been expressed in *E. coli*, showing varied yields and mostly antifungal activity [81,90,121]. An nsLTP heterologously produced in *E. coli*, isolated from dill (*A. graveolens, AgLTP*) had its antibacterial activity inhibited when tested against phytobacteria. However, it presented antifungal activity (IC50) between 20–40 µM (*Aspergillus niger*) and >40 µM (*Neurospora crassa*), even though it did not completely inhibit fungal growth when used at the highest concentration (40 µM) [78].

In some cases, the antimicrobial activity of small heterologously expressed proteins in *E. coli* may be compromised. This can occur due to scarce post-translational modifications at the end of heterologous production in prokaryotes, even with coupled fusion proteins that mimic these modifications. However, some heterologously expressed proteins have their antimicrobial activity optimized when they are bound to fusion proteins, as these can act as chaperones, facilitating AMP folding [122]. A similar approach was carried out by Cai et al. [120] when a protein isolated from ginseng (*Panax ginseng*) showed antifungal activity when fused to the target protein, inhibiting the growth of five fungi taxa (*Sclerotinia* sp., *Rhizoctonia solani*, *Phytophthora cactorum*, *Fusarium solani*, *Cylindrocarpon destructans*) in concentrations of 2.41, and 7.23 µM. In turn, the post-purified protein (without fusion protein) inhibited only one fungus (*Sclerotinia* sp.) in a concentration of 0.12 µM.

### 8.3. Expression in Yeast

Yeasts, as *Pichia pastoris*, have been considerably used in the production of heterologously expressed proteins due to their higher yield and most effective antimicrobial activity compared to *E. coli* [114]. This is due to the combination of properties that unite characteristics of prokaryotic (such as exponential growth) and of eukaryotic organisms, as the ability to carry out post-translational modifications [123]. In addition, the expression system in yeast has other advantages such as low endotoxin content compared to bacteria and, if the expression conditions are optimized, there may be a significant increase in the final yield. An example is the work by Pokoj et al. [124] which expressed an *nsLTP* (*Cor a. 8*) from a hazelnut (*Corylus avellana*) allergen in *P. pastoris*. The authors obtained a higher expression rate, followed by a final yield of 82 mg/L—clearly higher when compared to yield obtained in *E. coli* (0.3 mg/L)—when added an additional purification step using size exclusion chromatography in order to obtain a protein with a higher degree of purity and to favor protein folding.

In contrast, some studies faced some difficulties in *nsLTPs* recombinant expression, even in *P. pastoris*. This is the case of the work by Oeo-Santos et al. [112] where despite the isolation and identification of the primary sequence of an *nsLTP* extracted from olive (*Olea europaea*) pollen, a large number of expressed isoforms prevented its cloning and expression. Thus, it was necessary to modify the primary sequence of the isolated protein, as well as carry out identity and similarity analyzes comparing the altered protein with the initially isolated one. At the end, it was possible to heterologously produce the allergen from olive, obtaining a final yield of 1.5 mg/L. The recombinant protein was also subjected to a comparative structural analysis. Both proteins were not able to recover the native secondary structure when subjected to heat treatment. Finally, an extensive immunological characterization was performed comparing both proteins (natural and recombinant), and it was observed that both share most of the IgG and IgE epitopes, in addition to being effective in diagnosing allergy in vitro.

### 8.4. Purification and Yield of Expressed Products

Peptides can be chemically synthesized or produced through cellular machinery. This last process has been chosen in many works for its easy handling, low cost and rapid growth in cell density [125]. Among other hosts, fast-growing microorganisms such as *E. coli*, *S. cerevisiae* and *P. pastoris* can be used to produce proteins. Table 3 lists some nsLTPs produced and purified from heterologous expression.

Recombinant nsLTP sequences generally incorporate a carrier protein in its N-terminal portion, ensuring attenuation of its toxicity to the host organism and the protection of the peptide against degradation in the medium and increasing solubility [69,129]. Some perform additional functions such as glutathione-S-transferase (GST), used in pg-LTP, and maltose-binding protein (MBP) in rHev b 12, which also act as chaperones, thus promoting protein folding [121,126,130]. Some studies, however, have opted for thioredoxin in heterologous production, as it was the case of AgLTP, LcLTPs, Pru p. 3 and PsLTP1 expressed in *E. coli* [78,90,127].

The purification of heterologous products can start in two ways: precipitation of nsLTPs by ammonium sulfate or separation by column chromatography. Some nsLTPs were strategically fused with carrier proteins and/or tags for easy initial separation by matrix-specific chromatography. For unfused recombinant nsLTPs, precipitation was shown to be interesting as the first purification step. For example, Pokoj et al. [124] initially separated the unfused Cor a 8 in two precipitation steps: (1) initially it removed most of the unwanted proteins by adding ammonium sulfate at 50% saturation and then (2) the Cor a 8 was precipitated in 80% saturation.

Purification by chromatography is based on how recombinant nsLTPs were produced by the presence or absence of tags and carrier proteins. In the first case, histidines are strategically adopted as tags in recombinant nsLTPs due to their high affinity to metal ions. Thus, these proteins are initially separated by immobilized metal affinity chromatography (IMAC), being adsorbed, for example, on nickel resin [53,78,90,129]. Whereas, nsLTPs produced without tags and carrier proteins are separated by fast protein liquid chromatography (FPLC) in size exclusion (SEC) and ion exchange (IEC) modes [124]. In the end, optionally, all forms of nsLTPs are separated by RP-HPLC.

As shown in Table 3, most of the host systems used for the production of nsLTPs were *E. coli* cultures, although lower yields were observed in relation to the other systems. As evaluated in some works, the purification yield of nsLTPs expressed by *E. coli* ranged between 0.3 and 5 mg/L of host culture [53,78,90,124,126,127]. Choosing another producing microorganism is the alternative to achieve good yields. Pokoj et al. [124], for example, achieved a yield of 82 mg/L of Cor 8 through expression in the yeast *P. pastoris*, a value sixteen times higher than the highest yield achieved in *E. coli* [90]. The low yield of bacterial production may be related to its reduced cytoplasmic space, making difficult the formation of disulfide bridges. Besides, bacterial cellular machinery does not favor post-translational modifications [124].

## 9. PLTPs as Antimicrobial Agents

In recent decades bacteria acquired resistance to several drugs concerning different types of infection [131]. The resistance of these organisms to antibiotics is linked to continuous selective pressure as well as the development and improvement of survival strategies in response to the demand for synthesized antibiotics [132]. In this scenario, plants represent a significant source for the investigation of compounds with a broad spectrum of action to various virulent bacterial strains [133]. The routes through which the traffic of nsLTPs occurs are still being investigated [133]; however, several in vitro and in vivo antimicrobial activities have been attributed to these molecules, thus attracting the interest of industries focused on human health [134].

### 9.1. Biotechnological Applications on Plant Defense

Currently, it is known that the expression of genes encoding nsLTPs is associated with numerous biological processes, linked to plant development and stimulated by the presence of environmental stresses [135], thus the nsLTPs help for plant homeostasis, being active in several metabolic pathways [33,134]. As antimicrobial agents, their main strategy is to disturb the integrity and permeability of pathogens’ biological membranes [84]. Such an effect turns nsLTPs into promising candidates for drug development against human bacterial pathogens [24]. They are also important biotechnological targets for the plant, due to their involvement in several important physiological pathways, including energy source, plant defense against biotic and abiotic stresses, and cell signaling [67] besides their close relation to lipids [68].

Characterized as multifunctional proteins [23], the participation of nsLTPs in the adaptation of plants to biotic and abiotic stresses is one of the most relevant strategies that has been reported [32]. The understanding of the regulatory profile of these proteins favors work involving transgenic technologies and molecular improvement, benefiting cultivars of agronomic interest resistant to biotic and abiotic stresses [32,136].

#### 9.1.1. In Vitro Studies

Biological activity is one of the main issues that have been explored in nsLTPs, based on evidence of direct response to bacteria and fungi, making them molecules of choice against various plant diseases. This feature was confirmed from in vitro studies carried out with protein extracts of radish (*Raphanus sativus*), *A. thaliana* and spinach (*Spinacia oleracea*) [91,137], for instance.

Cammue et al. [77] isolated a potent nsLTP from onion seeds (*Ace-AMP1*) that was able to inhibit the growth of 12 tested fungi and Gram-positive bacteria (*Bacillus megaterium* and *Sarcina lutea*) at concentrations below 10 μg/mL. The antifungal activity of an *nsLTP* isolated from sunflower (*Helianthus annuus*) seeds was reported by Regente and Canal [138]. Also, a transgenic rice (*O. sativa* ssp. *indica*) expressing *nsLTP* from *Dahlia merckii* inhibited the in vitro growth of the main fungal and bacterial rice pathogens (*Magnaporthe grisea*, *Rhizoctonia solani* and *Xanthomonas oryzae*) [62].

The introduction of *LjAMP2* and *nsLTP* from *Leonurus japonicus* in Chinese White Poplar (*Populus tomentosa*) transgenic plants allowed an inhibitory in vitro activity against the fungal pathogens (*Alternaria alternata* and *Colletotrichum gloeosporioides*). In the same work, in vivo assays were performed, and the symptoms derived from the two fungi were lower in transgenic poplar plants compared to wild type [139].

#### 9.1.2. In Vivo Studies

nsLTPs play several roles during in vivo assays, including lipid exchange between cytoplasmic organelles and defense against pathogens [140]. Jung, Kim and Hwang [74] introduced a pepper (*C. annuum*) transgene (*CaLTP*) into *A. thaliana* plants inducing a faster plant development compared to wild type, in addition to conferring resistance to *Pseudomonas syringae* pv. *tomato* and *Botrytis cinerea*, besides tolerance to abiotic stresses (salt and drought).

A wheat *nsLTP* was introduced alone, and together with a barley chitinase, into agrobacterium-transformed carrot (*Daucus carota*) plants [141]. As a result, infection by leaf necrotrophic pathogens (*Alternaria radicícola* and *Botrytis cinerea*) was reduced from 90% to 95% in transformants with both proteins, compared to 40% to 50% in transgenic plants containing only the *nsLTP*, indicating that the interaction of nsLTPs with other proteins can improve their efficiency against pathogens.

Studies on the involvement of *nsLTPs* in tolerance to abiotic stresses bring promising results. For instance, *ZmLTP3* isolated from maize increased salt tolerance in *A. thaliana* transgenic plants compared to wild plants [142]. In another example Xu et al. [32] analyzed the functional potential of an *NtLTP4* isolated from *N. tabacum* in transgenic tobacco plants, observing different functional effects. First, an improvement in saturation and drought tolerance was observed. Then it was verified that *NtLTP4* transformed plants presented an increase of the transcription levels of the sodium exchanger gene NHX1, responsible for establishing ionic homeostasis, as well as HKT1 transporters, responsible for the homeostasis of Na^+^ and K^+^, thus alleviating the toxicity by Na^+^. The acquisition of *NtLTP4* also increased the expression of ROS scavenging enzymes under drought and salinity conditions. Finally, *NtLTP4* was able to interact with a member of the MAPK family, defined as a ‘wound-induced kinase’. This study highlighted the various functionalities associated with the de *nsLTPs* expression.

Considering the above, even though it is not completely clear how *nsLTPs* respond to a great demand for biological processes [67], the continuity of in vitro and in vivo studies figure as promising strategies, which demand specific issues underlying their function [111].

### 9.2. Biotechnology Applied to Human Health

Problems associated with bacterial resistance and infectious diseases are increasingly emerging and have led to a demand for new antimicrobial molecules with a broad spectrum of activity and few side effects [143]. Due to the scarcity of effective compounds, the use of last-resort cytotoxic drugs has been adopted, such as colistin, a drug based on an antimicrobial peptide. Its effectiveness has been seriously compromised by the spread of plasmids from the *mcr-1* gene [144]. In this scenario, new AMPs have been sought as promising alternatives to traditional antibiotics. Among them are nsLTPs, which have many functions, including antibacterial, antifungal, antiviral, antiproliferative activities, besides inhibiting some enzymes [23]. In this scenario, new AMPs have been sought as promising alternatives to traditional antibiotics. Among them are nsLTPs, which have the most varied functions, which include antibacterial, antifungal, antiviral, antiproliferative activities [136]. Desirable effects, as the formation of pores that alter the permeability of the pathogen’s membrane, have been reported for nsLTPs. The positive charge of these AMPs favors the binding to negatively charged molecules, such as phospholipids and lipopolysaccharides, resulting in cell death [145].

As stated, nsLTPs have been the focus of many antimicrobial studies against phytopathogenic microorganisms, but their effects against human pathogens through in vivo experiments remain poorly explored. Despite that, the ability of some nsLTPs to inhibit the growth of important human pathogenic microorganisms has drawn attention to its potential clinical use. As an example, *CmLTPs* isolated from *Chelidonium majus* showed strong broad-spectrum antibacterial activity against *Campylobacter jejuni*, *Listeria greyi* and *Clostridium perfringens* [146].

Souza et al. [24] observed the in vivo activity of McLTPs obtained from *Morinda citrifolia* seed extracts, with excellent potential to inhibit the growth of Gram-positive microorganisms of the genus *Staphylococcus* spp., and the ability to reduce biofilm formation by *S. aureus*. In turn, such activity was not observed against *Pseudomonas aeruginosa*, *E. coli* and *Enterococcus hirae*. When used synergistically with Oxacillin, McLTP has shown promising effects by increasing the sensitivity of *S. aureus* and *S. epidermidis* to the drug. Such findings confer less susceptibility to the microorganism and can lead to a reduction in therapeutic doses and a shorter duration of treatment. McLTPs can also play a protective effect during bacterial infections. Such activity may be related to in vivo results showing the reduction of pro-inflammatory cytokines, TNF-α, IL-6 and MCP-1, promoting a decrease in body weight loss, fever, leukocytosis and organ damage. These results confirm the report of Campos et al. [88] who demonstrated that McLTP has the ability to reduce cytokines related to the harmful effects of inflammation (TNF-α, IL-1 and IL-6), increasing the levels of anti-inflammatory cytokine IL-10 and decreasing the migration of neutrophils in vivo to the site of infection, without showing cytotoxic effects.

The effects of McLTP1 were also evaluated against edema formation and inflammatory cell infiltration in pancreatitis, where McLTP1 was able to reduce pancreatic edema, serum amylase, lipase levels and pancreatic and pulmonary myeloperoxidase, by increasing TNF -α, IL-1β and IL-6, reduced levels of IL-10, causing a decrease in pancreatic and pulmonary edema, decreasing pancreatic damage [88].

Systemic fungal infections are often underestimated but are life-threatening and associated with high mortality in immunocompromised individuals [147]. Antifungal agents used in current clinical practice suffer a progressive decline in their efficacy due to their low availability, low spectrum of action and high toxicity. Thus, there is an increasing need to obtain new active compounds against fungal infections that have wide availability [148]. For example, nsLTPs from seeds of *Canavalia ensiformes, Coffea canephora* and *Capsicum annuum*, demonstrated activity against *Candida tropicalis, S. cerevisiae, Schizosaccharomyces pombe* [149], *C. albicans* [83], *Fusarium oxysporum* [150], *S. cerevisiae, C. parapsilosis, Kluyveromyces marxiannus* and *C. guilliermondii* [85].

Another beneficial effect exhibited by nsLTPs is the antiproliferative inhibitory capacity of HIV-1 reverse transcriptase, in addition to inhibiting the proliferation of hepatoma cells MCF7 and breast cancer HepG2 [80]. The nsLTPs isolated from *Narcissus tazetta* showed a similar ability to inhibit human acute promyelocytic leukemia (HL-60) tumor cells besides antiviral activity against influenza A (H1N1) virus and respiratory syncytial virus (RSV) [151]. As revised by Perretti et al. [152], there is evidence of a role of nsLTPs in cancer-associated signal transduction cascades, as nsLTPs may interact with membrane contact sites, contributing to the generation of cell malignant phenotype associated with tumorigenesis in humans.

nsLTPs isolated from seeds of *Capsicum annuum*, *Coffea canephora* and *Vigna unguiculata* (VuLTP) inhibited the activity of human salivary α-amylases (HSAs) [153]. Many identified α-amylases have an active site composed of a certain negatively charged triplet of amino acids (Asp-Glu-Asp) whereby alpha-amylase inhibitor amino acids can physically interact with one or more residues of the triplet. Among the mentioned nsLTPs, VuLTP stands out for being rich in positively charged amino acid residues [65] capable of binding to HSA, obstructing the carbohydrate-binding cleft and making substrate fixation difficult [154]. Such α-amylase inhibition mechanism allows such peptides to be improved and applied in the prevention of postprandial carbohydrate digestion in humans, with potential benefits in the control of obesity [155].

In addition to the antimicrobial, anti-inflammatory and inhibitory activities, pharmacological studies have shown that nsLTPs have antinociceptive activity, making them a promising alternative to current drugs available on the market that demonstrates high toxicity and low efficacy. McLTP1 obtained from *M. citrifolia* seeds demonstrated pain-related modulatory activity in vivo and did not show neurological disorders such as locomotor activity, suggesting that the nsLTP antinociceptive response was highly selective [87].

Another action of interest involves the use of nsLTPs to provide chemical and physical stability to pharmacological compounds since drugs must maintain their properties during storage until their administration by the patient for therapeutic efficacy [153]. Due to the instability of many active principles, it is necessary to guarantee protection against oxidation, extreme conditions and proteolytic degradation. An alternative to face this problem is the use of transport systems that include proteins that adhere to ligands [156]. nsLTPs are classic transport proteins that can act by conjugating to hydrophobic molecules, including fatty acids [157], acetyl-CoA [5] and phosphatidyl glycerol [158] ensuring stability to the compound and facilitating the drug delivery system. nLTPs become good candidates due to their proven ability to bind lipids present in the skin such as oleic acid and sphingosine derivatives, thus being an interesting alternative when there is a need for drugs to penetrate the lipid membrane [156].

The thermodynamic stability of the interaction drug-nsLTP is fundamentally performed by hydrogen bonds, electrostatic interactions and van der Waals forces [159]. Such interactions can be established in several ways since distinct binding sites have already been observed in interactions with different nsLTPs. For instance, maize nsLTP I has a highly polar binding site on the protein surface. A second attachment site was reported in the hydrophobic cavity that, due to its flexibility, provides enough space to house phenyl-type molecules. In rice nsLTP II, a possible active site is present in a cluster of apolar residues close to the hydrophobic cavity conferring the capacity to interact with sterol-like molecules [148].

In addition to other drugs, nsLTPs can also act as transport systems linked to antibiotics, such as Amphotericin B, used in the treatment of systemic fungal infections. The chances of interaction between nsLTP and the membrane surface of this drug are high, since Amphotericin B is a bulky and rigid molecule, rich in hydrophobic portions and hydroxyl groups [159].

## 10. Toxicity, Allergenicity and Side Effects

As mentioned, nsLTPs are known as food allergens that have broad resistance to food processing as well as to the gastrointestinal environment [160] with many cases reported [161]. It is possible to decrease the immunogenicity of nsLTPs by incorporating these proteins into relevant structures such as lipid complexes. Thus, by changing the protein structure and IgE binding sites, the development of antibodies by the patient’s innate immune system can be provided, thus decreasing the allergic response [162]. These findings reinforce the application of nsLTPs in clinical contexts. However, since nsLTPs are of plant origin, they present significant allergen potential. These are the prevalent causes of primary allergy in adults in Mediterranean countries, where they induce the greatest number of food-dependent anaphylactic reactions, mainly from nuts [163] and other vegetables, as tomatoes [53].

During protein processing, new IgE epitopes are exposed, presenting potential new food allergens such as nsLTPs [164]. It is common for patients sensitized to nsLTPs to have allergies to different foods of plant origin, since the chances of nsLTPs causing cross-reactions among themselves are high, a condition called ‘nsLTP Syndrome’. It has also been observed that the interaction between nsLTPs and non-steroidal antibiotics (NSAIDs) can increase hypersensitivity to nsLTPs. Such antibiotics can act as co-factors in the clinical expression of food allergy, causing dysregulation of the epithelial barrier and higher permeability of the intestinal mucosa, increasing the exposure of the allergen to the patient’s immune system [165].

Furthermore, nsLTPs belong to the prolamine superfamily and have an α-helix motif maintained by disulfide bridges, providing high structural stability. This stability confers them the ability to resist proteolytic digestion and heat, thus acting as allergens even in cooked and processed foods [166]. Currently, the diagnosis of allergies is predominantly performed with skin tests using extracts. The purification or production of new nsLTPs is a first step to unravel the relationship between their allergenicity and structural characteristics and to enable the quantification of specific IgE antibodies for the development of new immunoassays and in vitro diagnosis of allergies [167].

Naturally, nsLTPs are present at different levels in various plant food sources. Higher levels of such molecules were reported in fruits of the Rosaceae family, such as apple, pear, cherries, etc. [168], but a literature review (Table 3) shows their prevalence in many other popular and largely consumed food sources. As told, most cases of allergenicity have been reported in the Mediterranean region and it would be interesting and opportune to assess whether dietary habits or genetic factors in the Mediterranean population are associated with this prevalence. As can be seen in Table 4, few studies address the problem in southern hemispheric populations (especially in the tropics). Such a lack of worldwide detailed studies of population allergenicity profiles deserves reflection, also biasing the data available for comparison.

In humans, plant nsLTPs are thought to contribute to some major food allergies or even plant pollen syndrome [188]. Rougé et al. [189], showed that these proteins were still present in processed tomato-derived products by testing positive in sensitive patients. Thus, the physicochemical characteristics and great stability of nsLTPs favor the maintenance of their allergenic and immunogenic motifs even after passing through the gastrointestinal tract, which provides an interaction with the epithelial immune system to induce sensitization and systemic symptoms [190]. The reported tolerance to degradation is believed to increase nsLTPs ability to potentiate allergic sensitization and increase the severity of allergic symptoms [188]. IgE-binding epitopes have been identified by Borges et al. [191], covering more than 40% of the surface of nsLTPs in plum, apple, apricot and peach (all Rosaceae family). Furthermore, the authors emphasized that the outer topography of several other nsLTPs consists of an extensive surface area occupied with IgE bonds in easily accessible regions of the molecule. Such accessibility is very different from those of other plant allergens that have a short coverage area by IgE-binding epitopes on the molecular surface. Eventually, hypoallergenic substances should be useful tools for immunotherapy for nsLTP-mediated allergies to Rosaceae fruits. Molecular approaches to allergy research and diagnosis are important for a better understanding of nsLTP allergy, also helping with appropriate patient counseling.

## 11. Patents and Products

**Figure 6:**
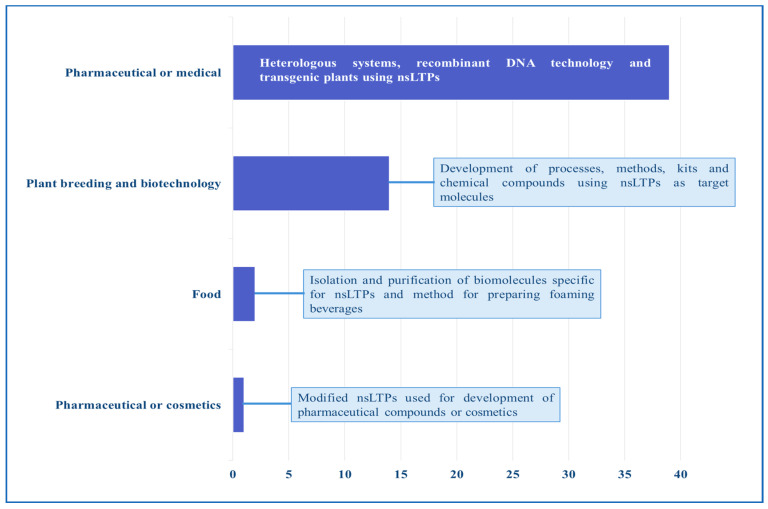
Patents and applications associated with nsLTPs (non-specific lipid-transfer protein) are available in the database of the US Patent and Trademark Office.

After a keyword search using the terms “lipid transfer protein” OR “LTP” in the title or abstract in the US Patent and Trademark Office database, 121 patents associated with the terms used were retrieved. After curation, 56 patents were considered hits for describing nsLTPs as target molecules in the described processes (Table S1), which were classified into four groups according to their applications (Figure 6).

The most representative group of patents referred to applications in the “Pharmaceutical or medical industry”, with 39 patents (Table S1) involving the development of processes, methods, diagnostic kits and replacement of chemical compounds used as nsLTPs inhibitors aiming application in the prevention and treatment of cardiovascular diseases or development of anti-allergic vaccines and immunotherapy of allergic diseases.

In turn, the group “Plant Breeding and Biotechnology” totaled 14 patents (Table S1) involving five plant species (cotton, barley, corn, soybean and wheat), with greater emphasis on the use of nsLTPs promoters (modified or not) to modulate the expression of genes of interest in cell lines, specific tissues/organs and transgenic plants aiming to introduce or enhance desirable agronomic characteristics by heterologous systems or recombinant DNA technology. In addition, some patents were also aimed at methods of production and use of regulatory molecules isolated from nsLTPs and their applications or methods for obtaining embryos cultivated in somatic plant cells using nsLTP analogs introduced into the culture medium in efficient concentrations to induce cell differentiation.

The third group of patents, related to the use of nsLTPs in the food industry, involves two patents that describe the isolation and purification of new monoclonal antibodies specific for nsLTPs that influence the quality of sparkling beverages and a method for producing sparkling beverages using an nsLTP from cereals as a foaming additive.

The fourth group of patents includes a single patent with application in the pharmaceutical or cosmetics industry, which reports a method for the production of a modified nsLTP by incorporating a lipophilic active substance for the development of drugs or cosmetics containing the modified LTP as an active ingredient.

## 12. Conclusions and Perspectives

LTPs (also named nsLTPs or pLTPs) are widely known for their antimicrobial activities (including antibacterial, antifungal, and antiviral), especially considering their strategy of disturbing pathogens’ membrane integrity. The fact that these AMPs are present and induced in most plant tissues, even in the absence of pathogen attack, deserves special attention as they strongly indicate the existence of other (still unexplored) roles for these molecules.

As shown throughout this review, a great deal of research has involved the analysis of the effects of nsLTPs in plants, in their native state or even through transgenic strategies.

Applications in human health have been recognized, especially considering the antimicrobial action. However, additional effects have been predicted, some of interest for drug development, including activities such as antinociceptive, proapoptotic, induction of cell signaling under stress, ROS scavenging (antioxidant), antiproliferative effects on cancer cells (hepatoma, breast cancer and promyelocytic leukemia), besides α-amylase inhibition (with potential use to control obesity).

Considering viruses, previous studies included antiproliferative activity against HIV-1 and H1N1 viruses. Since both are RNA viruses, tests with SARS-CoV-2 (the etiologic agent of COVID-19) would be desirable.

Another interesting potential concerns the high stability of these peptides, which has led to the consideration of using nsLTPs to provide chemical and physical stability to pharmacological compounds.

In several roles mentioned, the association of the mentioned activities with the known antimicrobial effect of nsLTPs would be desirable. One application not yet considered involves food protection and increase of shelf life. However, there is still a long way to go, and confirmation of the proposed activities and testing in mammalian and human models are mandatory. The reported allergenicity of nsLTPs also requires attention, demanding studies applying peptide engineering, in order to reduce allergenic regions, without significant loss of the reported functions.

Finally, it should be noted that the search for nsLTPs in omics databases—recommended and described here—should bring new insights into these molecules, including a better understanding of their evolution and the emergence of new functions, with impacts on the understanding of their biotechnological applications in plant improvement and human health.

## Figures and Tables

**Table 1:** Survey of stress situations, putative functions and expression of ns LTPs in different plant species.

Plant Taxa	Expressed under/Putative Function	Experimental Background	References
*Arabidopsis thaliana*	Plant development, biotic stress, abiotic stress	Microarray, RT-qPCR	[62,72]
*Brassica* spp.	Abiotic stress, biotic stress	EST, vector cloning, RNA-Seq	[55,69]
*Capsicum annuum*	Abiotic stress, biotic stress, molecular signaling	RNA gel blot, RT-qPCR	[73,74]
*Glycine max*	Abiotic stress, biotic stress	In silico SuperSAGE tags screening	[52]
*Oryza sativa*	Abiotic stress, plant development	RT-qPCR	[75]
*Saccharum* spp.	Abiotic stress, as signaling molecule	RT-qPCR	[60]
*Solanum lycopersicum*	Plant development	RNA-Seq/RT-qPCR	[54]
*Triticum* spp.	Biotic stress, abiotic stress	RT-qPCR	[25,29]
*Vigna unguiculata*	Abiotic stress, biotic stress, plant development	Northern blot, vector cloning	[66,76]
*Zea mays*	Biotic stress	RT-qPCR	[71]

**Table 2:** Natural occurring nsLTPs identified in plants, including main isolation steps (type of extraction, precipitation and chromatography) as well as recorded biological activities.

Species	nsLTP Name	Source	Extraction	Precipitation	Chromatography (or Other)	Biological Activity	References
*Allium cepa*	Ace-AMP1	Seed	Buffer solution	ammonium sulfate (85%) ^a^	ion exchange and RP-HPLC ^b^	antifungal and antibacterial	[77]
*Anethum graveolens*	Ag-LTP	Aerial parts	Buffer solution	-	ion exchange and RP-HPLC	antifungal *	[78]
*Beta vulgaris*	IWF5	Leaf	Acid	-	ion exchange and RP-HPLC	antifungal	[79]
*Brassica campestris*	*Brassica campestris* nsLTP	Seed	Not mentioned	-	ion exchange, affinity and gel filtration	antifungal	[80]
*Brassica rapa*	BrLTP2.1	Nectar	-	-	1-D SDS-PAGE	antifungal *	[81]
*Chelidonium majus*	CmLTP 9.5	Root latex	Buffer solution	isoelectric point	1-D SDS-PAGE	antibacterial **	[82]
*Coffea canefora*	Cc-LTP1	Seed	Buffer solution	ammonium sulfate (90%)	ion exchange and RP-HPLC	α-amylase inhibitor and antifungal	[83]
	Cc-LTP2	Seed	Acid	-	ion exchange and RP-HPLC	antifungal and antibacterial	[84]
*Cuminum cyminum*	Cumin nsLTP1	Seed	Buffer solution	ammonium sulfate (72%)	gel filtration and RP-HPLC	-	[85]
*Helianthus annus*	HA-AP10	Seed	Buffer solution	ammonium sulfate (80%)	gel filtration, ion exchange and RP-HPLC	antifungal	[86]
*Morinda citrifolia*	McLTP1	Seed	Buffer solution	-	gel filtration and RP-HPLC	antibacterial, anti-inflammatory ^c^, lethal sepsis prevention ^c^ and antinociceptive ^c^	[24,87,88]
*Phaseolus mungo*	mung bean nsLTP	Seed	Buffer solution	ammonium sulfate (80%)	ion exchange	antifungal and antibacterial	[89]
*Pisum sativum*	Ps-LTP1	Seed	Buffer solution	-	ion exchange and RP-HPLC	antifungal ^d^, antibacterial ^d^ and allergen ^d^	[90]
*Raphanus sativus*	radish ns-LTP-like	Seed	Not mentioned	ammonium sulfate (30–70%)	ion exchange and RP-HPLC	antifungal	[91]
*Trachyspermum ammi*	ajwain nsLTP1	Seed	buffer solution	ammonium sulfate (80%)	gel filtration and RP-HPLC	-	[92]

^a^ relative saturation value of ammonium sulfate; ^b^ RP-HPLC = reverse-phase high-performance liquid chromatography; ^c^ tested in mice; ^d^ tested in native and recombinant nsLTP; * biological activity from recombinant nsLTP; ** biological activity from protein fraction of nsLTP + other proteins, but not from the isolated peptide.

**Table 3:** Recombinant LTPs produced by different heterologous expression systems, including tested biological activity and final post-purification yield.

Origin Species	Host System	Express nsLTP	Biological Activity	Yield (mg.L^−1^ of Host Culture)	Reference
*Anethum graveolens*	*Escherichia coli*	His8-TrxL-Ag-LTP	Antifungal	1.5	[78]
*Brassica rapa*	*Escherichia coli*	T7-BrLTP2.1-His6	Antifungal	Not informed	[81]
*Corylus avellana*	*Escherichia coli*	His6-Cor a 8	Allergen	0.3	[125]
	*Pichia pastoris*	Cor a. 8	Allergen	82	[125]
*Hevea brasiliensis*	*Escherichia coli*	MBP-rHev b 12	Allergen	4	[126]
*Lens culinaris*	*Escherichia coli*	His8-TrxL-Lc-LTP1	Antifungal, antibacterial, allergen	3	[127]
	*Escherichia coli*	His8-TrxL-Lc-LTP3	Antifungal, antibacterial, allergen	5	[127]
*Prunus persica*	*Escherichia coli*	His8-TrxL-Pru p 3	Antifungal, antibacterial, allergen	4	[127]
*Linum usitatissimum*	*Saccharomyces cerevisiae*	(His tag) *LuLTP_Ls*1 e *LuLTP_Ls*4	Antibacterial	Not informed	[128]
*Panax ginseng*	*Escherichia coli*	GST-pgLTP-His6	Antifungal	Not informed	[121]
*Pisum sativum*	*Escherichia coli*	His8-TrxL-Ps-LTP1	Antifungal, antibacterial, allergen	5	[90]
*Solanum lycopersicum*	*Escherichia coli*	(His tag) Sola l 3	-	0.5	[53]

**Table 4:** Allergenic nsLTPs identified in different foods, with reported geographic distribution.

Food Source	Taxon	nsLTP Allergen	Reported Geographical Areas	Sources
Apple	*Malus domestica* (Rosaceae)	Mald 3	Mainly southern Europe, also northern Europe and Australia	[169,170]
Asparagus	*Aparagus officinalis* (Asparagaceae)	Aspao 1	Mainly southern Europe	[171]
Cabbage	*Brassica oleracea* (Brassicaceae)	Brao 3	Mainly southern Europe	[172]
Chestnut	*Castanea sativa* (Fagaceae)	Cass 8	Mainly southern Europe, also USA	[173]
Grape	*Vitis vinifera* (Vitaceae)	Vitv 1	Mainly southern Europe, also Australia and Germany	[168]
Green bean	*Phaseolus vulgaris* (Leguminosae)	Phav 3	Mainly southern Europe	[174]
Hazelnut	*Corylus avellana* (Betulaceae)	Cora 8	Mainly southern Europe, also Switzerland and Denmark	[175]
Kiwi	* Actinidia deliciosa * (Actinidiaceae)	Actd10	Mainly southern Europe	[176]
Lentil	*Lens culinaris* (Leguminosae)	Lenc 3	Mainly southern Europe	[177]
Lettuce	*Lactuca sativa* (Asteraceae)	Lacs 1	Mainly southern Europe	[178]
Mandarin	*Citrus reticulata* (Rutaceae)	Citr 3	Mainly southern Europe	[179]
White Mustard	*Sinapis alba* (Brassicaceae)	Sina 3	Mainly southern Europe	[180]
Oranges	*Citrus* spp. (Rutaceae)	Cits 3	Mainly southern Europe	[181]
Peanut	*Arachis hypogaea* (Leguminosae)	Arah 9	Mainly southern Europe, also USA	[182,183]
Pomegranate	*Punica granatum* (Punicaceae)	Pung 1	Mainly southern Europe	[184]
Tomato	*Solanum lycopersicon* (Solanaceae)	Lyce 3	Mainly southern Europe	[185]
Walnut	*Juglans regia* (Juglandaceae)	Jugr 3	Mainly southern Europe, also USA	[186]
Wheat	*Triticum aestivum* (Poaceae)	Tria 14	Mainly southern Europe	[187]

## Supplementary Materials

The following are available online at https://www.mdpi.com/article/10.3390/antibiotics10111281/s1, Table S1: Patents regarding nsLTPs (non-specific lipid-transfer protein) deposited in the US Patent and Trademark Office (USPTO).

## Abbreviations

pLTPs	Plant lipid transfer proteins
nsLTPs	Non-specific lipid transfer proteins
AMPs	Antimicrobial peptides
PR-proteins	Pathogenesis-related proteins
LRR	Rich domain in leucine
PK	Kinase protein
SAR	Systemic acquired resistance
BID	Pro-apoptotic protein
IAA	Acetic acid-2-indole
ABA	Abscisic acid
JA	Jasmonic acid
kDa	Kilo dalton
Cys	Cysteine
H	Helix
L	Loop
PCR	Polymerase chain reaction
RP-HPLC	Reverse-phase high-performance liquid chromatography
μM	Micromolar
pI	Isoelectric point
pH	Hydrogen potential
MAPK	Mitogen-activated protein kinase
ROS	Oxygen-reactive species
GST	Glutathione s-transferase
MBP	Maltose binding protein
NHX1	Sodium/hydrogen exchanger agent
HKT1	Sodium carrier
AINEs	Non-steroidal antibiotics
USPTO	United States Patent and Trademark Office
