# Supplementary material for: Lipid Transfer Proteins (LTPs)—Structure, Diversity and Roles beyond Antimicrobial Activity
Source: Antibiotics (Basel). 2021 Oct 21;10(11):1281. doi: 10.3390/antibiotics10111281 (PMC8615156; doi:10.3390/antibiotics10111281)
Supplement: Supplementary file 1 [file antibiotics-10-01281-s001.zip › antibiotics-1344501-supplementary.pdf]

Table S1. Patents regarding nsLTPs (non-specific lipid-transfer protein) deposited in the US Patent and Trademark Office (USPTO) *.

Accession Number	Deposit Date	Description	Application
5,585,235	December, 1999	Fluorescent method for measuring the activity of CETP or Microsomal Transfer Protein (MTP) and a device that determines the activity of CETP or MTP by the use of a newly synthesized donor particle without regard to the presence of colored or otherwise interfering factors. More particularly, includes a synthetic particle for determination of neutral LTP (CETP or MTP) activity with provisions to correct for interference that would normally occur during the measurement of the activity	Medicine and Pharmaceutical industry
6,448,295	September, 2002	Substituted polycyclic aryl and heteroaryl tertiary-heteroalkylamine compounds useful as inhibitors of CETP; LTP-I, and compounds, compositions and methods for treating cardiovascular diseases. Preferred tertiary-heteroalkylamine compounds are substituted for *N-fused-phenyl-N-benzyl aminoalcohols	Medicine and Pharmaceutical industry
6,451,823	September, 2002	Substituted polycyclic aryl and heteroaryl tertiary-heteroalkylamine compounds useful as inhibitors of CETP; LTP-I, and compounds, compositions and methods for treating cardiovascular diseases. Preferred tertiary-heteroalkylamine compounds are substituted for N-phenoxy-N-phenyl aminoalcohols	Medicine and Pharmaceutical industry
6,451,830	September, 2002	Substituted polycyclic aryl and heteroaryl tertiary-heteroalkylamine compounds useful as inhibitors of CETP; LTP-I, and compounds, compositions and methods for treating cardiovascular diseases. Preferred tertiary-heteroalkylamine compounds are substituted for N,N-disubstituted non-fused heterocyclo amines	Medicine and Pharmaceutical industry
6,455,519	September, 2002	Substituted polycyclic aryl and heteroaryl tertiary-heteroalkylamine compounds useful as inhibitors of CETP; LTP-I, and compounds, compositions and methods for treating cardiovascular diseases. Preferred tertiary-heteroalkylamine compounds are substituted for N,N- disubstituted fused-heterocyclo amines	Medicine and Pharmaceutical industry
6,458,803	October,2002	Substituted polycyclic aryl and heteroaryl tertiary-heteroalkylamine compounds useful as inhibitors of CETP; LTP-I, and compounds, compositions and methods for treating cardiovascular diseases. Preferred tertiary-heteroalkylamine compounds are substituted for N-phenyl-N-heteroaralkyl aminoalcohols	Medicine and Pharmaceutical industry
6,458,849	October, 2002	Substituted polycyclic aryl and heteroaryl tertiary-heteroalkyamine compounds useful as inhibitors of CETP; LTP-I and compounds, compositions and methods for treating cardiovascular diseases. Preferred tertiary-heteroalkylamine compounds are substituted for N,N-disubstituted mercapto amines	Medicine and Pharmaceutical industry
6,458,852	October, 2002	Substituted polycyclic aryl and heteroaryl tertiary-heteroalkylamine compounds useful as inhibitors of CETP; plasma LTP-I and compounds, compositions and methods for treating cardiovascular diseases. Preferred tertiary-heteroalkylamine compounds are substituted for N,N- bis-phenyl aminoalcohols	Medicine and Pharmaceutical industry
6,462,092	October,2002	Substituted polycyclic aryl and heteroaryl tertiary-heteroalkylamine compounds useful as inhibitors of CETP; plasma LTP-I, and compounds, compositions and methods for treating cardiovascular diseases. Preferred tertiary-heteroalkylamine compounds are substituted for N,N-disubstituted reverse aminoalcohols	Medicine and Pharmaceutical industry

6,476,057	November, 2002	Substituted polycyclic aryl and heteroaryl tertiary-heteroalkylamine compounds useful as inhibitors of CETP; plasma LTP-I, and compounds, compositions and methods for treating cardiovascular diseases. Preferred tertiary-heteroalkylamine compounds are substituted for N,N-disubstituted cycloalkyl aminoalcohols	Medicine and Pharmaceutical industry
6,476,057	November, 2002	Substituted polycyclic aryl and heteroaryl tertiary-heteroalkylamine compounds useful as inhibitors of CETP; plasma LTP-I, and compounds, compositions and methods for treating cardiovascular diseases. Preferred tertiary-heteroalkylamine compounds are substituted for N,N-disubstituted cycloalkyl aminoalcohols	Medicine and Pharmaceutical industry
6,476,075	November, 2002	Substituted polycyclic aryl and heteroaryl tertiary-heteroalkylamine compounds useful as inhibitors of CETP; plasma LTP-I, and compounds, compositions and methods for treating cardiovascular diseases. Preferred tertiary-heteroalkylamine compounds are substituted for N,N-bis-benzyl aminoalcohols	Medicine and Pharmaceutical industry
6,479,552	November,2002	Substituted polycyclic aryl and heteroaryl tertiary-heteroalkylamine compounds useful as inhibitors of CETP; plasma LTP-I, and compounds, compositions and methods for treating cardiovascular diseases. Preferred tertiary-heteroalkylamine compounds are substituted for N,N-disubstituted diamines	Medicine and Pharmaceutical industry
6,482,862	November, 2002	Substituted aromatic polycyclic tertiary-heteroalkylamine compounds as inhibitors of CETP; plasma LTP-I, and compounds, compositions and methods for treating cardiovascular diseases. Preferred substituted aromatic polycyclic tertiary-heteroalkylamine compounds are substituted for N-benzyl-N-phenyl aminoalcohols	Medicine and Pharmaceutical industry
6,521,607	February, 2003	Substituted aryl and heteroaryl (R)-Chiral Halogenated 1-Substitutedamino-(n+1)-Alkanol compounds useful as inhibitors of CETP; plasma LTP-I, and compounds, compositions and methods for treating cardiovascular diseases. Preferred (R)-Chiral 1-Subsbitutedamino-(n+1)-Alkanol compounds are substituted for (R)-Chiral N-phenoxy N-phenyl aminoalcohols	Medicine and Pharmaceutical industry
6,544,974	April, 2003	Substituted aryl and heteroaryl (R)-Chiral Halogenated 1-Substitutedamino-(n+1)-Alkanol compounds useful as inhibitors of CETP; plasma LTP-I, and compounds, compositions and methods for treating cardiovascular diseases. Preferred (R)-Chiral 1-Substitutedamino(n+1)-Alkanol compounds are substituted for (R)-Chiral fused heterocyclic amino compounds	Medicine and Pharmaceutical industry
6,583,183	June, 2003	Substituted polycyclic aryl and heteroary tertiary-heteroalkylamine compounds useful as inhibitors of CETP; plasma LTP-I, and compounds, compositions and methods for treating cardiovascular diseases. Preferred tertiary-heteroalkylamine compounds are substituted for N-phenyl-N-fused-benzyl aminoalcohols	Medicine and Pharmaceutical industry
6,586,433	July, 2003	Substituted polycyclic aryl and heteroary tertiary-heteroalkylamine compounds useful as inhibitors of CETP; plasma LTP-I, and compounds, compositions and methods for treating cardiovascular diseases. Preferred tertiary-heteroalkylamine compounds are substituted for N-heteroaryl-N-phenyl aminoalcohols	Medicine and Pharmaceutical industry
6,677,341	January, 2004	Substituted aryl and heteroaryl (R)-Chiral Halogenated 1-Substitutedamino-(n+1)-Alkanol compounds useful as inhibitors of CETP; plasma LTP-I, and compounds, compositions and methods for treating cardiovascular diseases. Preferred (R)-Chiral 1-Substitutedamino-(n+1)-Alkanol compounds are substituted for (R)-Chiral heteroaryl aminoalcohols	Medicine and Pharmaceutical industry

6,677,353	January, 2004	Substituted polycyclic aryl and heteroary tertiary-heteroalkylamine compounds useful as inhibitors of CETP; plasma LTP-I, and compounds, compositions and methods for treating cardiovascular diseases. Preferred tertiary-heteroalkylamine compounds are substituted for N-phenyl-N-heteroaralkyl aminoalcohols	Medicine and Pharmaceutical industry
6,677,375	January,2004	Substituted polycyclic aryl and heteroary tertiary-heteroalkylamine compounds useful as inhibitors of CETP; plasma LTP-I, and compounds, compositions and methods for treating cardiovascular diseases. Preferred tertiary-heteroalkylamine compounds are substituted for N,N-bis-benzyl aminoalcohols	Medicine and Pharmaceutical industry
6,677,379	January,2004	Substituted polycyclic aryl and heteroary tertiary-heteroalkylamine compounds useful as inhibitors of CETP; plasma LTP-I, and compounds, compositions and methods for treating cardiovascular diseases. Preferred tertiary-heteroalkylamine compounds are substituted for N,N-disubstituted diamines	Medicine and Pharmaceutical industry
6,677,380	January,2004	Substituted polycyclic aryl and heteroaryl tertiary-heteroalkylamine compounds useful as inhibitors of CETP; plasma LTP-I, and compounds, compositions and methods for treating cardiovascular diseases. Preferred tertiary-heteroalkylamine compounds are substituted for N,N-disubstituted mercapto amines	Medicine and Pharmaceutical industry
6,677,382	January,2004	Substituted polycyclic aryl and heteroaryl tertiary-heteroalkylamine compounds useful as inhibitors of CETP; plasma LTP-I, and compounds, compositions and methods for treating cardiovascular diseases. Preferred tertiary-heteroalkylamine compounds are substituted for N,N-bis-phenyl aminoalcohols	Medicine and Pharmaceutical industry
6,683,099	January,2004	Substituted polycyclic aryl and heteroaryl tertiary-heteroalkylamine compounds useful as inhibitors of CETP; plasma LTP-I, and compounds, compositions and methods for treating cardiovascular diseases. Preferred tertiary-heteroalkylamine compounds are substituted for N,N-disubstituted cycloalkyl aminoalcohols	Medicine and Pharmaceutical industry
6,683,113	January,2004	Substituted aryl and heteroaryl (R)-Chiral Halogenated 1-Substitutedamino-(n+1)-Alkanol compounds useful as inhibitors of CETP; plasma LTP –I, and compounds, compositions and methods for treating cardiovascular diseases. Preferred (R)-Chiral 1-Substitutedamino-(n+1)-Alkanol compounds are substituted for (R)-Chiral N,N-bis-benzyl aminoalcohols	Medicine and Pharmaceutical industry
6,696,435	February, 2004	Substituted polycyclic aryl and heteroaryl tertiary-heteroalkylamine compounds useful as inhibitors of CETP; plasma LTP-I, and compounds, compositions and methods for treating cardiovascular diseases. Preferred tertiary-heteroalkylamine compounds are substituted for N,N-disubstituted fused-heterocyclo amines	Medicine and Pharmaceutical industry
6,696,472	February, 2004	Substituted polycyclic aryl and heteroaryl tertiary-heteroalkylamine compounds useful as inhibitors of CETP; plasma LTP-I, and compounds, compositions and methods for treating cardiovascular diseases. Preferred tertiary-heteroalkylamine compounds are substituted for N-phenoxy-N-phenyl aminoalcohols	Medicine and Pharmaceutical industry
6,699,898	March, 2004	Substituted polycyclic aryl and heteroaryl tertiary-heteroalkylamine compounds useful as inhibitors of CETP; plasma LTP-I, and compounds, compositions and methods for treating cardiovascular diseases. Preferred tertiary-heteroalkylamine compounds are substituted for N,N-disubstituted non-fused heterocyclo amines	Medicine and Pharmaceutical industry

6,710,089	March, 2004	Substituted polycyclic aryl and heteroaryl tertiary-heteroalkylamine compounds useful as inhibitors of CETP; plasma LTP-I, and compounds, compositions and methods for treating cardiovascular diseases. Preferred tertiary-heteroalkylamine compounds are substituted for N-fused-phenyl-N-benzyl aminoalcohols	Medicine and Pharmaceutical industry
6,723,752	April, 2004	Substituted aryl and heteroaryl (R)-Chiral Halogenated 1-Substitutedamino-(n+1)-Alkanol compounds useful as inhibitors of CETP; plasma LTP-I, and compounds, compositions and methods for treating cardiovascular diseases. Preferred (R)-Chiral 1-Substitutedamino-(n+1)-Alkanol compounds are substituted for (R)-Chiral N-benzyl-N-phenyl aminoalcohols	Medicine and Pharmaceutical industry
6,723,753	April, 2004	Substituted polycyclic aryl and heteroaryl tertiary-heteroalkylamine compounds useful as inhibitors of CETP; plasma LTP-I, and compounds, compositions and methods for treating cardiovascular diseases. Preferred tertiary-heteroalkylamine compounds are substituted for N-benzyl-N-phenyl aminoalcohols	Medicine and Pharmaceutical industry
6,765,023	July, 2004	Substituted polycyclic aryl and heteroaryl tertiary-heteroalkylamine compounds useful as inhibitors of CETP; plasma LTP-I, and compounds, compositions and methods for treating cardiovascular diseases. Preferred tertiary-heteroalkylamine compounds are substituted for N,N-disubstituted reverse aminoalcohols	Medicine and Pharmaceutical industry
6,787,570	September, 2004	Substituted N-Alkyl/Alkenyl/Cycloalkyl/Heterocycyl N-Aryl/Heteroaryl tertiary-Heteroalkylamine compounds useful as inhibitors of CETP; plasma LTP-I, and compounds, compositions and methods for treating cardiovascular diseases. Preferred tertiary-heteroalkylamine compounds are substituted for N-cycloalkyl N-benzyl aminoalcohols	Medicine and Pharmaceutical industry
6,803,388	October, 2004	Substituted aryl and heteroaryl (R)-Chiral Halogenated 1-Substitutedamino-(n+1)-Alkanol for (R)-Chiral N,N-bis-phenyl aminoalcohols compound useful as inhibitors of CETP; plasma LTP-I, and compounds, compositions and methods for treating cardiovascular diseases. Preferred (R)-Chiral 1-Substitutedamino-(n+1)-Alkanol compounds are substituted (R)-Chiral N,N-bis-phenyl aminoalcohols	Medicine and Pharmaceutical industry
6,861,561	March, 2005	Substituted aromatic polycyclic tertiary-heteroalkylamine compounds useful as inhibitors of CETP; plasma LTP-I, and compounds, compositions and methods for treating cardiovascular diseases	Medicine and Pharmaceutical industry
7,122,536	October, 2006	Substituted aryl and heteroaryl (R)-Chiral Halogenated 1-Substitutedamino-(n+1)-Alkanol compounds useful as inhibitors of CETP; plasma LTP-I, and compounds, compositions and methods for treating cardiovascular diseases	Medicine and Pharmaceutical industry
7,253,211	August, 2007	Substituted aryl and heteroaryl (R)-Chiral Halogenated 1-Substitutedamino-(n+1)-Alkanol compounds useful as inhibitors of CETP; plasma LTP-I, and compounds, compositions and methods for treating cardiovascular diseases	Medicine and Pharmaceutical industry
7,700,658	April, 2010	Substituted aryl and heteroaryl (R)-Chiral Halogenated 1-Substitutedamino-(n+1)-Alkanol compounds useful as inhibitors of Cholesteryl Ester Transfer Protein (CETP; plasma LTP-I) and compounds, compositions and methods for treating cardiovascular diseases	Medicine and Pharmaceutical industry
10,526,365	January, 2020	Method of making and a modified allergen having reduced allergenicity and preserved immunogenicity compared to corresponding native allergenic material. The raw allergenic material is selected from the group consisting of DP mites extract, Der p1, ovalbumin and LTP	Medicine and Pharmaceutical industry

5,525,716	June, 1996	*In vivo* expression in the aleurone cells of a cereal of a conjugate. The conjugate comprises a gene of interest and a LTP2 gene promoter. The conjugate is stably integrated within the cereal's genomic DNA	Plant breeding or biotechnological inferences
5,792,933	August,1998	Cotton fiber-specific LTP its amino acid sequence, its protein-encoding DNA sequence, and its 5' flanking sequence. In addition, the promoter and upstream sequences of two other LTP. Finally, methods for utilizing these sequences to express genes of interest in Gossypium hirsutum L., in a fiber-specific fashion	Plant breeding or biotechnological inferences
5,914,270	June, 1999	Method for promoting differentiation of cells in culture. At least LTP or LTP analog is introduced into a culture medium at a concentration that is effective for obtaining differentiation of cells in the culture medium	Plant breeding or biotechnological inferences
6,031,152	February,2000	An expression system for at least the aleurone cells of a developing caryopsis or for at least the scutellar epithelial tissue or vascular tissue of a germinating seedling or developing grain or plant. The expression system comprises a gene promoter fused to a GOI. In a preferred embodiment the expression system comprises the GOI fused to a modified LTP1 promoter	Plant breeding or biotechnological inferences
6,939,958	September, 2005	Regulatory region obtained from a wheat aleurone gene LTPW1. This region contains mutations or deletions of this regulatory region, useful to express heterologous genes of interest (GOI) within aleurone plant cells. Furthermore, a truncated LTPW1 regulatory region that exhibits constitutive activity with monocot and dicot plants and is also directed to vectors comprising these regulatory regions operatively linked with a heterologous GOI, as well as plant cell cultures and transgenic plants comprising these vectors	Plant breeding or biotechnological inferences
7,417,179	August, 2008	Method for producing a plant having a modified epidermal outgrowth structure such as a trichome or root hair, comprising of transforming a plant or plant cell with a D-type cyclin gene operably linked to epidermis-preferred promoters (e.g., LTP3, GL2, GORK, MIP-MOD, BLEC4, WAXD9 and MtENOD12) and regenerating a plant from the transformed plant or plant cell	Plant breeding or biotechnological inferences
7,550,579	June, 2009	Novel nucleotide sequences for a pericarp-preferred promoter and terminator isolated from the maize LTP1 coding region and method for expressing a heterologous nucleotide sequence in a plant using the regulatory sequences	Plant breeding or biotechnological inferences
7,851,614	December, 2010	Novel nucleotide sequences for a pericarp-preferred promoter and terminator isolated from the maize LTP1 coding region and method for expressing a heterologous nucleotide sequence in a plant using the regulatory sequences	Plant breeding or biotechnological inferences
7,897,746	March, 2011	Novel nucleotide sequences for a pericarp-preferred promoter and terminator isolated from the maize LTP1 coding region and method for expressing a heterologous nucleotide sequence in a plant using the regulatory sequences	Plant breeding or biotechnological inferences
8,143,475	March, 2012	Promoters of a soybean LTP4 and fragments thereof and their use in promoting the expression of one or more heterologous nucleic acid fragments in plants	Plant breeding or biotechnological inferences
8,158,858	April, 2012	Promoters of a soybean LTP1 and fragments thereof and their use in promoting the expression of one or more heterologous nucleic acid fragments in plants	Plant breeding or biotechnological inferences
8,759,612	June, 2014	Gene expression regulatory sequences from soybean LTP2, fragments thereof, and their use in promoting the expression of one or more heterologous nucleic acid fragments in plants	Plant breeding or biotechnological inferences

8,859,746	October, 2014	Non-coding regulatory element polynucleotide molecules isolated from the LTP gene of *Oryza sativa* and useful for expressing transgenes in plants	Plant breeding or biotechnological inferences
10,544,423	January, 2020	Recombinant fiber-selective promoter region comprising a DNA molecule comprising a fiber specificity region of a cotton Lipid Transfer Protein (LTP) gene promoter, operably linked to a specific DNA molecule and use thereof to increase fiber-selective expression of products of interest in cotton fiber	Plant breeding or biotechnological inferences
5,993,865	November,1999	Beverage containing proteins and/or peptides and is characterized in that it contains Cereal-LT and/or homologues as herein defined and/or a modified Cereal-LTP fraction obtainable from the Cereal-LTP and/or homologues by heating, boiling and/or mashing the Cereal-LTP and/or homologues in water at a pH between 3 and 7. Additionally, a method for preparing it and a use of a foam-forming additive	Food industry
6,423,546	July, 2002	Novel monoclonal antibodies reactive with LTPs typically found in foaming beverages. More specifically, the present invention relates to novel monoclonal antibodies raised against the native and denatured forms of barley LTP1, and an assay for determining the content of said proteins in foaming beverages at various stages of their production	Food industry
5,324,821	June, 1994	Method of preparing a lipoprotein modified by incorporation of a lipophilic active substance as well as a pharmaceutical or cosmetic composition containing a lipoprotein modified and at least one LTP, in this way as an active ingredient	Pharmaceutical or cosmetic industry

* Patents identified from the search using the term “Lipid transfer protein” or “LTP” in the title or abstract.

Legend for abbreviations: Cholesteryl Ester Transfer Protein (CETP); Gene of interest (GOI); Lipid Transfer Protein (LTP); Microsomal Transfer Protein (MTP).
